# Two years at the editor’s desk: reflections on community, diversity and impact

**DOI:** 10.1093/conphys/coag040

**Published:** 2026-06-18

**Authors:** Andrea Fuller

**Affiliations:** Department of Physiology, University of the Witwatersrand, 7 York Road, Parktown, Johannesburg, 2193, South Africa

As I conclude my second year as Editor-in-Chief of *Conservation Physiology*, I look back on a challenging but enjoyable period, shaped by my commitment to maintaining the Journal’s high standards while fostering a community grounded in diversity, impactful science and strong support for early-career researchers (Fuller, 2024). Through the dedicated efforts of Bridget O’Boyle in the editorial office, the editorial board and teams at Oxford University Press (OUP) and the Society for Experimental Biology (SEB), the Journal is well positioned for the future, and I am pleased to share some recent developments.

## Diversity shaping our science and our community

Over the past 2 years, our editorial board has welcomed new members whose diverse expertise, geographic representation and perspectives have strengthened both our review processes and our strategic direction. During this time, we also said goodbye to several long-serving associate editors, whose contributions shaped not only the Journal but also the broader field of conservation physiology. To support continued renewal, all editors now serve 4-year terms, renewable once, ensuring a regular infusion of new ideas and stewardship.

Sean Tomlinson, based in Australia, assumed the role of Plant Science editor in 2024 and is working with a newly appointed team of associate editors to encourage submissions that address the role of physiology in guiding plant conservation. This focus is evident in our recent special issue on ‘Traits and Measurements in Plant Conservation’ (see Tomlinson, 2025). Our current call for papers on ‘Applications of Mechanistic Modelling in Biodiversity Conservation’ continues this emphasis, inviting work that uses mechanistic models to understand and address biodiversity conservation challenges, across all taxa, including plants.

There is diversity not only in the geographical locations and areas of expertise represented on our editorial board, but also in the submissions we receive. While the majority of submissions still come from authors in the USA, Canada and Australia, we are now receiving an increasing number of manuscripts from a much broader range of countries, including countries such as Argentina, Ecuador, Mexico, Bangladesh, India, Malaysia, China, Norway, Germany and Kenya. This growing geographic spread reflects the expanding global reach of the Journal and the field of conservation physiology.

In terms of submissions by taxa, fish continue to be most frequently represented in the Journal, but terrestrial and marine mammals are now close behind. Overall, we receive manuscripts on the conservation physiology of an impressively broad range of species. Recent publications span various physiological systems in organisms as diverse as green turtles off the coasts of Costa Rica (Bruno *et al.,* 2025) and Chile (Álvarez-Varas *et al.,* 2025), golden star tunicates in the Northeast Pacific (Tobias *et al.,* 2025), honeybees in Italy (Ferrari *et al.,* 2024), giant salmonfly nymphs from Montana (Verhille *et al.,* 2024), parrot-beaked dwarf tortoises in South Africa (Garcia and Clusella-Trullas, 2025), thick-billed murres (Eby *et al.,* 2024) in Canada and the seeds of South Korean (Gu *et al.,* 2024) and Australian (Rajapakshe *et al.,* 2024) plants. The breadth of submissions means that we rely heavily on the wide-ranging expertise of our editorial board and our reviewers.

## Championing the next generation of conservation physiologists

Championing the next generation of conservation physiologists is vital for both the discipline and the ecosystems we seek to protect. Early-career researchers typically bring fresh perspectives and innovative approaches to understand how species respond to stressors imposed by climate change, habitat disturbance and other human-driven pressures. Yet the path for these emerging scientists is demanding. Limited funding, high expectations and the need to balance rigorous mechanistic research, often in a field setting, with applied conservation outcomes can be overwhelming.

To help empower early-career researchers, *Conservation Physiology* has implemented several initiatives. In partnership with the SEB, we started a new webinar series, ‘Community Conversations’, aimed primarily at early-career researchers wishing to building a career in conservation physiology or related fields. The first two webinars, given by two of the world’s foremost experts in the field, Professors Steven Cooke (emeritus editor of *Conservation Physiology*) and Brian Helmuth (associate editor, *Conservation Physiology*), were well received. Steven Cooke reflected on co-production, a crucial approach for generating actionable science to inform conservation (Cooke *et al.,* 2025), while Brian Helmuth discussed escaping anthropocentric constraints to better enact conservation strategies in coastal ecosystems. We then held a webinar, based on our recent editorials (Clements *et al.,* 2025; Tomlinson *et al.,* 2026), to discuss constructive ways for writing and responding to peer reviews. The webinar series will continue to cover topics related to the field of conservation physiology, as well as sessions that aim to strengthen early-career skills in areas such as publishing, science communication and collaboration. Please follow our Bluesky or X account for updates on webinars, or check the SEB website’s Event Calendar. 

*Conservation Physiology* encourages contributions from early-career researchers, and we are delighted that students have been first authors on roughly half of all articles submitted over the past 2 years. To further promote their contributions, we now give preference to featuring the work of early-career researchers in our series ‘Conservation Physiology in Action’. These short articles, which themselves are written by other early-career researchers with guidance from experienced mentors, place conservation physiology research in a broader and more accessible context, and highlight the importance of the research for conservation and management. Among the Journal’s most-read articles this year is Banousse (2026), which reveals how shrinking ice is affecting polar bear pregnancies, based on the original work by McGeachy *et al.* (2025).

We also recently launched an editorial internship programme at *Conservation Physiology*, designed specifically for early-career researchers. This initiative provides hands-on experience of the editorial process, offering valuable insights into manuscript handling and peer review. In this first year, three appointments have been made: Rodolfo de Oliveira Anderson, Léa Lorrain-Soligon and Jeremy De Bonville. We look forward to welcoming more rising stars in our field and giving them the opportunity to learn how the Journal operates and how our editorial and review systems support high-quality science.

## A society-based journal investing in society

*Conservation Physiology* is a society-based journal, owned jointly by the SEB and OUP. As an Open Access journal, the Journal relies on authors to pay an article processing charge (APC) to cover the real costs of producing and maintaining the Journal, except where the authors qualify for a waiver or discount (low- and middle-income countries) or Open Access agreements through their institutions or consortia (also known as Read and Publish agreements). SEB members qualify for a discount on the APC, and we are pleased that the SEB has been able to increase that discount from 10 to 20% recently.

*Conservation Physiology* operates on a non-profit basis, such that any income accrued by the SEB is reinvested into the scientific community. In 2025, as in other years, the SEB reinvested their income into numerous events, grants, outreach, policy and engagement programmes, as well as career development programmes (Table 1). Many of these initiatives supported scientists in the Global South and early-career researchers. Two of the postdoctoral fellows in my own laboratory benefitted through travel grants to attend the SEB Annual Conference held in Antwerp in 2025. This reinvestment by the SEB contributes to strengthening the discipline of conservation physiology, as well as fostering networking and collaboration, and facilitating impactful science more broadly.

**Table 1:** Actions supported by SEB in 2025

**Support type**	**Examples**
Events	1. Helped fund the SEB’s 2025 Conference in Antwerp, Belgium, attracting over 800 delegates to 39 scientific sessions and 9 workshops. 2. Organised the PEPG Field Techniques Workshop in Portugal, with 92 attendees. 3. Helped fund the Journal of Experimental Botany’s 75th Anniversary Conference in Edinburgh, Scotland, with over 100 delegates.
Outreach	1. Delivered the first SEB podcast. 2. Partnered with Native Scientists to fund 10 heritage-language outreach interventions across five countries, reaching 178 migrant children and providing public engagement training open to all SEB members. 3. Developed new public engagement resources, including a Minecraft-based ecology teaching tool, and supported SEB members to design and deliver their own outreach activities. 4. Attended a wide range of public engagement events, including science festivals and school workshops.
Career development	1. Ran a series of career workshops for early-career researchers, covering research impact, peer review, scientific communication, media engagement, interdisciplinary collaboration and academic publishing. 2. Sponsored 20 places in the Voice of Young Science programme, supporting researchers who are inspired and motivated to take responsibility for the public conversation about science. 3. Launched a new webinar series called Community Conversations, supporting early-career researchers wishing to build careers in conservation physiology and related fields. 4. Ran the second iteration of the SEB mentoring scheme, supporting 33 mentoring partnerships.
Policy and engagement	1. Established a new partnership with the All-Party Parliamentary Group on Diversity and Inclusion in STEM and appointed two SEB member representatives who actively contributed to reports and roundtables informing UK STEM policy. 2. Strengthened the SEB’s external engagement through new strategic partnerships with the Federation of Experimental Biology Societies and the Brazilian Society of Plant Physiology, creating opportunities for joint activities, journal collaboration and reciprocal member benefits. 3. Sponsored the Global Plant Council to promote plant biology research and its importance to the public and policymakers. 4. The SEB’s Awards Nomination Task Force continued to help broaden the diversity of SEB award nominees and published a peer-reviewed article in JOSHA Journal summarising the group’s first-year outcomes.
Membership	1. Expanded the SEB membership to 1389 members across more than 55 countries. 2. Achieved significant student and early-career researcher members, who now represent over 60% of the total membership. 3. Increased membership of the SEB’s SIGs by 31%. 4. Established an Early-Career Researcher Working Group to ensure membership benefits are relevant to the community.
Grants	1. Awarded 51 travel grants totalling £16 810, enabling students and early-career researchers to attend the annual SEB conference. 2. Financially supported 23 small conferences. 3. Supported 11 projects through the SEB Outreach and Engagement Grant including hands-on school workshops in Nigeria, science festival installations in the UK, sustainable agriculture training for smallholder farmers in Kenya and a space plant biology interactive lab in Italy. 4. Awarded the SEB Educational Research Grant to support seven high-quality projects across the UK, Italy, South Africa, Uganda and Nigeria. These grants supported projects on inclusive laboratory design, transferable skills development in biology curricula, virtual learning environments, team-based learning and global best practice in experimental biology teaching. 5. Awarded four SEB Diversity Grants to support projects on inclusive teaching in the USA, equitable conference participation in South Africa, Indigenous research partnerships in Mongolia and the Black in Plant Science Conference 2025 (UK).

**Figure 1:**
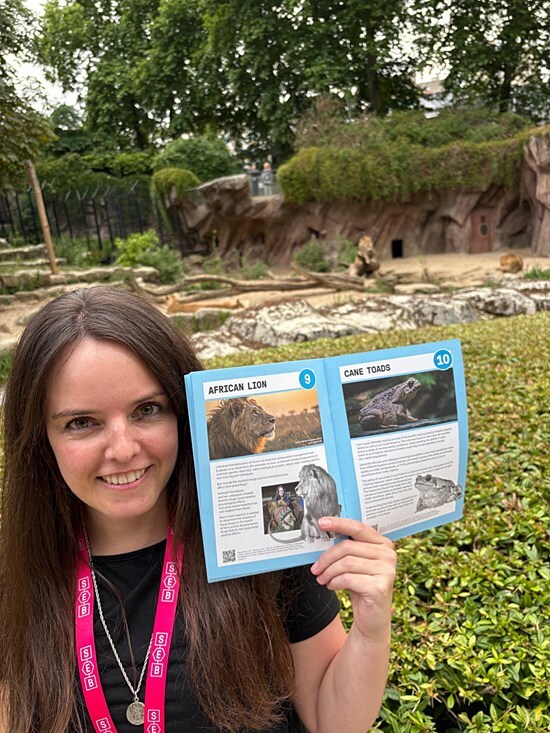
Ashleigh Donaldson, a postdoctoral fellow at the University of the Witwatersrand in South Africa, displays a feature on her doctoral work on African lions (Donaldson *et al.,* 2023) in our booklet ‘Exploring Antwerp Zoo’, whilst standing in front of the lion enclosure at Antwerp Zoo, at the 2025 SEB Annual Conference. Ashleigh also serves the Journal as Editor of the ‘Conservation Physiology in Action’ series (Photo credit: Andrea Fuller)

The SEB also supports the discipline of conservation physiology through various other initiatives. At the SEB Annual Conference in Antwerp, we worked with the SEB to produce a booklet to showcase how research from *Conservation Physiology* is helping to protect wild populations of the Zoo’s animals and plants (Fig. 1). The Annual Conference also hosts workshops and symposia in the area of conservation physiology each year. In 2025, we held both a workshop and symposium on the topic of biologging in conservation physiology, with that focused interest resulting in a special issue on ‘Insights and Tools from Biologging for Conservation Physiology’. We also arranged a symposium on ‘Shared Challenges and Diverse Approaches to Physiology in Conservation Across Taxonomic Boundaries’, and a workshop on peer review.

The SEB further supports our discipline through its Animal Section, and the Conservation Physiology Special Interest Group (SIG). This group, which can be joined through the SEB website, aims to bring together physiologists, ecologists and conservation biologists who have an interest in assessing and predicting the impacts of current and future human-induced environmental change on organisms, through its online discussion forum and other activities.

## Towards an impactful future in conservation physiology

We aim to advance *Conservation Physiology* by strengthening its role as a bridge between mechanistic physiology and applied conservation decision-making, while remaining committed to excellence, transparency, ethics, integrity and mutual respect (Cooke *et al.,* 2022). Increasingly, biologists are identifying as conservation physiologists, reflecting a growing alignment between studies of organismal function and conservation objectives. Our Journal continues to grow in usage (as reflected by total item requests), alongside improvements in the author experience, including shorter times to first decision and more rapid publication following acceptance. We are actively soliciting invited reviews from current and emerging leaders in the field, and look forward to seeing the impact not only through journal metrics, but also through meaningful real-world conservation outcomes.

None of our efforts to grow the Journal would be possible without the dedication of our editorial board and reviewers. Most importantly, our continued success depends on our authors. A strong future for conservation physiology requires a commitment to supporting people as fully as we support their science, and we hope our new initiatives contribute to that goal. We welcome engagement from both current and prospective authors and invite you to submit your work, share your ideas and join us in shaping the next chapter of *Conservation Physiology*.
